# Development of an Evaluation Index System for Health Recommender Systems Based on the Health Technology Assessment Framework: Cross-Sectional Delphi Study

**DOI:** 10.2196/79997

**Published:** 2025-12-22

**Authors:** Yue Sun, Shijie Hou, Siye Chen, Minmin Leng, Zhiwen Wang

**Affiliations:** 1 School of Nursing, Health Science Center, Xi'an Jiaotong University, Xi'an, Shaanxi Province, China; 2 School of Nursing, Peking University, 38 Xueyuan Road, Haidian District, Beijing, 100191, China, 86 15901566817, 86 01082802248; 3 Department of Nursing, Shandong Provincial Hospital Affiliated to Shandong First Medical University, Jinan, Shandong Province, China

**Keywords:** health recommender systems, health technology assessment evaluation framework, HTA evaluation framework, evaluation index system, Delphi survey, artificial intelligence, AI

## Abstract

**Background:**

Health recommender systems (HRSs) are digital platforms designed to deliver personalized health information, resources, and interventions tailored to users’ specific needs. However, existing evaluations of HRSs largely focus on algorithmic performance, with limited scientific evidence supporting user-centered assessment approaches and insufficiently defined evaluation metrics. Moreover, no unified or scientifically validated framework currently exists for evaluating these systems, resulting in limited cross-study comparability and constraining regulatory and implementation decision-making.

**Objective:**

This study aimed to develop a comprehensive, consensus-based evaluation index system for HRSs grounded in the health technology assessment (HTA) framework.

**Methods:**

This cross-sectional study used a 2-round Delphi process conducted with 18 experts comprising clinicians, digital health researchers, and policymakers who possessed relevant professional experience and domain knowledge in HRSs. The age range of the experts was between 30 and 58 years, with 67% (n=12) of them possessing over 10 years of professional experience. On the basis of literature analysis and HTA principles, a preliminary indicator set comprising 5 primary and 16 secondary indicators was constructed. Experts rated the importance of each indicator using a 5-point Likert scale and provided qualitative suggestions for refinement. After the Delphi process, the analytic hierarchy process was applied to determine indicator weights and assess consistency.

**Results:**

The Delphi survey reached full participation in the first round (18/18, 100%) and maintained an 88.9% (16/18) response rate in the second round. The final evaluation index system of HRSs contained 5 first-level indicators (performance, effectiveness, safety, economy, and social appropriateness) and 18 second-level indicators. The mean importance scores of the second-level indicators ranged from 4.25 (SD 0.45) to 5.00 (SD 0.00), with coefficients of variation between 0.000 and 0.220. Among the first-level indicators, safety received the highest weight (0.289), followed by social appropriateness (0.251), effectiveness (0.193), performance (0.136), and economy (0.132).

**Conclusions:**

This study presents an evaluation index system for HRSs grounded in the HTA framework and validated through expert consensus. The resulting framework not only provides actionable guidance for the design, optimization, and implementation of HRSs but also fills a methodological gap in the field by offering quantifiable, hierarchical evaluation indicators with validated weighting. Future research will involve iterative refinement and empirical validation of the system in real-world deployment settings, thereby enabling continuous improvement and facilitating the establishment of unified evaluation standards for HRS research and practice.

## Introduction

As technology evolves, new ways to implement tailored interventions are being adopted, and researchers and policymakers require access to appropriate tools to assess the design and suitability for use of these interventions [1]. One such innovative approach to computer-based tailored health interventions is recommender systems (RSs) [2]. Health RSs (HRSs), specialized digital platforms or software applications designed to recommend personalized information, resources, or interventions relevant to the user’s specific health needs, are now available to address this need [3]. A scoping review of the current literature identified 51 studies on HRSs covering a range of health domains, including general health promotion, lifestyle, generic health service, and others [4]. These HRSs use sophisticated data analytic techniques, integrating machine learning algorithms and artificial intelligence frameworks to process and interpret diverse user-specific datasets, including medical histories, behavioral patterns, and individual preferences. By synthesizing these multifaceted data sources, HRSs generate personalized recommendations, targeted resources, and customized interventions tailored to the unique needs of each user [5]. Given that the information provided by HRSs can have a significant impact on individuals’ health decisions, it is essential to conduct comprehensive evaluations before their widespread adoption. Such assessments not only enable developers to refine the design and functionality of these applications but also establish a scientific foundation for evaluating their effectiveness and ensuring their safe and reliable use [6,7].

In 2018, Moshi et al [8] highlighted that the health technology assessment (HTA) evaluation framework then in use was inadequate for delivering holistic assessments of mobile health solutions in a clinical environment. Since then, the field has advanced significantly, driven by the growing development and application of HTA evaluation frameworks for assessing digital health technologies (DHTs), especially mobile health [9,10]. As a health technology framework, HTA considerations encompass effectiveness, safety, and cost-effectiveness, as well as patient- and organizational-level factors, while also addressing ethical, social, and legal issues [11]. Furthermore, they include the technical characteristics of DHTs [12]. A scoping review of HRS research [4] found that most studies focus on performance metrics, with only a limited number of HRS studies incorporating evaluations of user participation, health outcomes, user acceptance, or feasibility. Key dimensions such as safety, economic impact, and social appropriateness have been largely overlooked. For example, these studies are either narrowly focused on specific system dimensions (eg, recommendation techniques) or health-related outcome measures [13-18] or they provide only a general overview of HRSs without giving adequate emphasis to evaluation [5,19]. De Croon et al [19] reviewed existing research related to HRSs and found that there is a lack of scientific evidence on user-centered evaluation approaches and that other metric parameters are ambiguous in terms of HRS evaluation and the feasibility of the organizational management of this technology. For example, the definition of performance varied across studies. Torrent-Fontbona and Lopez [20] used the amount of time in the glycemic target range by reducing the time below the target as a performance metric, whereas Cho et al [21] reported performance based on precision and recall. Given the heterogeneity in evaluation methods, it is necessary to develop a comprehensive and scientifically sound evaluation tool to provide reference and guidance for HRSs.

Accordingly, this study consisted of 2 main phases: first, summarizing and developing the evaluation indicators by reviewing the literature within the HTA framework and, second, identifying specific indicators and determining their weights through a Delphi expert consultation process and the analytic hierarchy process (AHP). Adopting an HTA-based evaluation indicator system could provide robust support for more systematic and in-depth assessment and offer guidance for the design, optimization, and implementation of HRSs in the health care domain [11].

## Methods

### Study Design

This study used a Delphi method [22,23], a widely recognized approach for selecting quality indicators in health care. The Delphi method does not prescribe a fixed number of rounds. Previous studies have consistently shown that 2 to 3 iterations are typically sufficient to reach consensus [24]. The process concludes when consensus is achieved on the topics under discussion. This study was designed and reported following the Delphi Studies in Social and Health Sciences–Recommendations for an Interdisciplinary Standardized Reporting framework [25], which provides a consensus-based methodological and reporting standard for Delphi research.

This study comprised 2 rounds of questionnaires administered to an expert panel via email, conducted in accordance with established methodological guidelines for Delphi surveys. The process consisted of two key stages: (1) developing a preliminary set of potential evaluation indicators along with a conceptual framework and secondary-level metrics for HRSs through a systematic literature review and (2) conducting a Delphi survey to prioritize and reach consensus on the primary and secondary evaluation indicators for HRSs. The specific process is illustrated in Textbox 1.

Textbox 1. **Summary of the 4-phase process used to develop the evaluation framework for the health recommender system, including expert panel selection, literature review, questionnaire preparation, and the Delphi–analytic hierarchy process procedure.**

**Phase 1: expert panel**

- Members held a postgraduate degree or higher, possessed a midlevel professional title or above, or had a minimum of 10 years of relevant work experience.
- Members were engaged in clinical medicine, clinical nursing, public health management, health-related government departments, or information technology related to recommender systems.

**Phase 2: literature review**

- Databases included PubMed, Embase, Web of Science, ACM, IEEE Xplore, ScienceDirect, CNKI, and Wanfang.
- Professional organization websites such as the World Health Organization and UNESCO were consulted.

**Phase 3: questionnaire preparation**

- The focus was on 5 aspects: technical characteristics, effectiveness, safety, economic considerations, and social appropriateness.
- The importance of each indicator was measured by its relevance to health recommender system performance, rated on a Likert scale from 1 to 5 (1=“not important”; 5=“very important”).

**Phase 4: Delphi method and the analytic hierarchy process**

- The reliability of the expert consultation was assessed through the expert positive coefficient, the authority coefficient, and the degree of opinion concentration and coordination.
- Using the Saaty scale, a judgment matrix was constructed, followed by hierarchical ranking and consistency testing.

### Participants

The selection criteria for experts were established as follows: (1) holding a postgraduate degree or higher, possessing a mid-level (or above) professional title, or having at least 10 years of relevant work experience; (2) being actively engaged in fields such as health IT, HTA, public health management, clinical medicine, nursing, or health-related government agencies, with substantial expertise and a thorough understanding of the research topic; and (3) voluntarily agreeing to participate in the study. Individuals lacking familiarity with HRSs were excluded from the selection process. A total of 20 eligible experts were invited via email, and 18 (90%) agreed to participate in the study.

### Questionnaire Preparation

This study, based on the HTA evaluation framework, aimed to develop a preliminary hierarchical framework for the evaluation indicator system of HRSs. Drawing on previous assessments of emerging health technologies, the evaluation focused on 5 key domains: technical characteristics, effectiveness, safety, economy, and social appropriateness, as shown in Table 1.

**Table 1.** The five-dimensional measurement framework for health recommender systems based on the health technology assessment model.

Aspect	Definition
Technical characteristics	Refers to the system’s effectiveness and reliability in delivering stable, high-quality recommendations.
Effectiveness	Refers to the ability of the health recommender system to achieve its intended objectives in real-world applications, such as promoting behavior change or improving health outcomes.
Safety	Refers to the system’s capacity to ensure user privacy, data security, operational stability, and overall user safety throughout its use.
Economy	Refers to the economic feasibility and cost-effectiveness of the health recommender system while fulfilling its functional requirements.
Social appropriateness	Refers to the degree to which the health recommender system aligns with sociocultural norms, ethical principles, and legal regulations, and is acceptable to users and society.

The following 8 electronic databases were searched on October 3, 2022: PubMed, Embase, Web of Science, ACM, IEEE Xplore, ScienceDirect, China National Knowledge Infrastructure, and Wanfang. Electronic searches were conducted using the following keywords: (“recommender systems”) OR (“recommender system”) OR (“recommendation systems”) OR (“recommendation system”) AND (health OR patient OR patients). To identify additional studies, we screened professional organization websites, including the World Health Organization, UNESCO, the Ministry of Health of the People’s Republic of China, the National Health Commission of China, and other organizational or governmental websites.

Potential studies including competency indicators were extracted and screened by 2 reviewers according to the following criteria: (1) studies that described or implemented HRSs with a primary focus on improving health and included an evaluation of the HRSs and (2) studies published in English or Chinese. In cases of uncertainty regarding the inclusion of a study, the research team held discussions to reach a consensus. On the basis of the analysis of the literature, a preliminary evaluation index system for HRSs was developed comprising 5 primary indicators (dimensions) and 16 secondary indicators.

The preliminary evaluation index system for HRSs was operationalized into a Delphi-based survey instrument. Within this questionnaire, the importance of each indicator was defined as its relevance in assessing the overall performance of HRSs, quantified using a Likert scale from 1 to 5 (1=“not important at all”; 5=“very important”). Additionally, the survey included open-ended questions to allow domain experts to provide feedback on the existing indicators and suggest additional indicators they deemed essential for inclusion.

### Delphi Method and the AHP

In the initial phase of expert consultations, a 5-point Likert scale was used by the panel of experts to evaluate and score the significance of each indicator, with an additional open-ended section for suggesting modifications. Upon the conclusion of this phase, the criteria for item retention were delineated as follows: an item’s mean importance score must surpass 4.0, the coefficient of variation (CV) must not exceed 0.25, and the selection rate for the highest score of 5 must be above 20% [25]. During the subsequent round of consultations, the anonymized results from the first round were disseminated among the experts, and the consultation form was augmented with a section for the allocation of index weights. In the final round, a select group of experts assigned weights to the index system, and the AHP was used to ascertain the weight values for the finalized evaluation index system [26]. This system is hierarchically organized into 3 strata: the goal layer, which encapsulates the overarching objective of the HRS evaluation; the criterion layer, encompassing the principal evaluation dimensions; and the indicator layer, comprising specific metrics. Experts conducted pairwise comparisons of indicators within the same stratum using a scale from 1 to 9 to quantify their relative importance (with 1 denoting equal importance and 9 signifying extreme importance), thereby constructing the judgment matrix [27].

### Index Screening and Result Statistics

The collected data were processed using Microsoft Excel before being input into a database for further analysis. Data analysis was conducted using Microsoft Excel 2021 and SPSS (version 26.0; IBM Corp). Descriptive statistics were used to calculate the mean, SD, and CV of the importance and feasibility of each indicator.

The reliability of the expert consultation was assessed through the expert positive coefficient, the expert authority coefficient (Cr), the degree of expert opinion concentration, and the degree of expert opinion coordination [28]. The expert positive coefficient was represented by the questionnaire response rate. The Cr was used to reflect the authority of expert opinions, calculated based on expert judgment (Ca) and their familiarity with the dimensions and items (Cs) using the formula Cr = (Ca + Cs)/2. The Kendall coefficient of concordance (*W*) and the CV were used to measure the degree of consensus among experts. A higher value of *W* indicates a stronger level of consensus among experts.

The weights of the indicators were determined using the AHP [29]. Through assigning importance values to each indicator, we established the Saaty scale, constructed the judgment matrix, and conducted a hierarchical ranking and consistency test. The consistency ratio is calculated to assess the judgment matrix. When the consistency ratio value is less than 0.10, it indicates that the judgment matrix exhibits satisfactory consistency and does not require further adjustments.

### Ethical Considerations

Ethics approval was obtained from the Biomedical Ethics Committee of Peking University (IRB00001052-22042). All participants provided informed consent through an electronic registration form where they were clearly informed that their responses would remain confidential and be used solely for research purposes. Participants were notified of their right to withdraw from the study at any time without penalty.

To ensure confidentiality and adhere to ethical standards, the questionnaire was administered anonymously. Only participants’ email addresses were collected for follow-up purposes, and these were stored separately from all demographic and survey data. All responses were deidentified during data processing, sharing of interim results between Delphi rounds, and final reporting. No personally identifiable information is present in this manuscript.

Access to identifiable data was restricted exclusively to the research team, and all data were securely stored within institutional systems. No compensation was provided to participants for their involvement in the study.

## Results

### Overview

**Figure 1.**
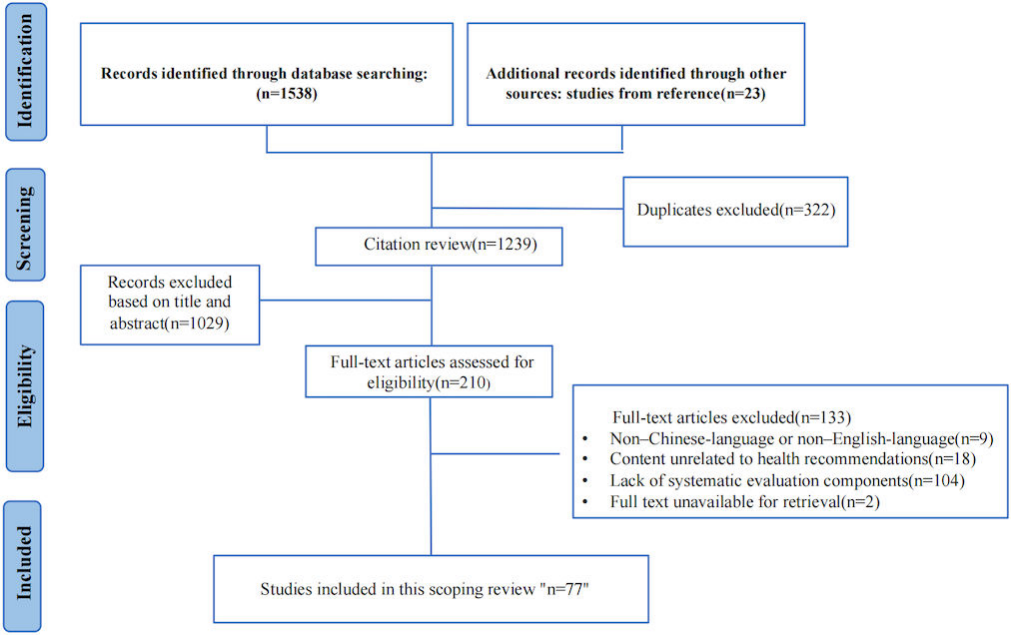
The process of study identification and selection. Flow diagram illustrating the literature search, screening, eligibility assessment, and final inclusion of studies used to inform the development of the health recommender system evaluation index system.

The initial literature search yielded a total of 1538 articles. After removing duplicates, of the 1538 articles, 1239 (80.6%) were screened by title and abstract for eligibility. Of these 1239 articles, 77 (6.2%) studies related to the evaluation of HRSs were included in the analysis. Figure 1 provides an overview of the identification and selection of studies at different stages of the screening process. The included studies were systematically organized based on the identified HTA evaluation framework, as detailed in Table S1 in Multimedia Appendix 1.

### Basic Information on the Experts

A total of 18 experts participated in the first round of the Delphi consultation. These experts were primarily engaged in fields such as clinical medicine, clinical nursing, public health management, health-related government departments, and IT related to RSs. The age range of the experts was between 30 and 58 years, with 67% (12/18) of them possessing over 10 years of professional experience. All experts (18/18, 100%) held a master’s degree or higher, and 72% (13/18) held senior professional titles (Table 2).

**Table 2.** Demographic characteristics of the 18 experts participating in the 2-round Delphi study (N=18).

Variable	Participants, n (%)
Age (y)	
30-39	10 (56)
40-49	6 (33)
≥50	2 (11)
Work experience (y)	
<10	6 (33)
10-19	8 (44)
20-29	4 (22)
Highest degree	
Master’s	9 (50)
PhD	9 (50)
Professional title	
Intermediate grade title	1 (5)
Deputy senior grade title	4 (22)
Senior grade title	13 (72)
Field of expertise	
Clinical medicine	5 (27)
Clinical nursing	4 (22)
Public health management	3 (16)
Health-related government departments	2 (11)
IT related to recommender systems	4 (22)

### Analysis of Expert Participation

#### Expert Positive Coefficient

Expert participation was assessed using the response rate and the proportion of experts who provided written feedback. In the first Delphi round, all distributed questionnaires were returned (18/18, 100% response rate) and an expert enthusiasm coefficient of 1.00. In the second round, 89% (16/18) of the questionnaires were returned. The retention rate across rounds was 89% (16/18) and an expert enthusiasm coefficient of 0.89.

#### The Cr Measure

Expert authority was assessed using the authority coefficient (Cr), defined as Cr = (Ca + Cs) / 2. In round 1, the mean Cr was 0.883, derived from a judgment coefficient (Ca) of 0.933 and a familiarity coefficient (Cs) of 0.833. In round 2, the mean Cr was 0.884, based on a Ca of 0.940 and a Cs of 0.827. All Cr values exceeded the commonly accepted threshold of 0.7, indicating a consistently high level of expert authority across both rounds.

#### The Degree of Expert Opinion Concentration

The degree of expert opinion concentration was assessed using the Kendall coefficient of concordance (*W*). In round 1, *W* was 0.281 for dimensions and 0.282 for items (c²=25.254 and 121.806; *P*<.001). In round 2, the values were 0.360 and 0.236 (c²=23.067 and 83.096; *P*<.001).

### Results of the Delphi Survey

#### First Round

The evaluation index system comprised 16 items across 5 dimensions. For the primary indicators, the mean importance scores ranged from 4.39 (SD 0.59) to 5.00 (SD 0.00), with CVs ranging from 0.000 to 0.172. All primary indicators met the inclusion criteria and were retained without modification. For the secondary indicators, the CVs ranged from 0.000 to 0.173, and the mean importance scores ranged from 4.17 (SD 0.60) to 5.00 (SD 0.00). Detailed results for dimensions and items are presented in Tables S2 and S3 in Multimedia Appendix 1 and Table 3.

**Table 3.** Adjustments to competency indicators for health recommender systems after round 1 of the Delphi survey.

Primary indicators	Secondary indicators	Adjustment
1. Performance	1.1 Accuracy (retained) 1.2 Content coverage (retained) 1.3 Result diversity (retained) 1.4 User trust (retained) 1.5 System robustness (retained)	Added “1.6 System response efficiency”
2. Effectiveness	2.1 Health behavior outcomes (modified) 2.2 Quality of life impact (modified) 2.3 Health service efficiency (modified)	Added “2.1 Health behavior” Merged 2.1 and 2.2 as “2.2 Health outcome” Modified 2.3 to “2.3 Quality and efficiency of health services”
3. Safety	3.1 Clinical safety (retained) 3.2 Technical application safety (retained)	No adjustments
4. Economy	4.1 Patient cost-effectiveness (modified) 4.2 Institutional cost-effectiveness (modified) 4.3 Social benefits (retained)	Modified 4.1 to “4.1 Patient-level economy” Modified 4.2 to “4.2 Institutional-level economy”
5. Social appropriateness	5.1 Data privacy protection (deleted) 5.2 Legal and policy compliance (modified) 5.3 Acceptability (retained)	Replaced 5.1 with “5.1 Ethicality” Modified 5.2 to “5.2 Policy suitability” Added “5.4 Feasibility”

a Not applicable.

#### Second Round

For the primary indicators, the mean importance scores ranged from 4.31 (SD 0.59) to 4.94 (SD 0.37), with CVs ranging from 0.00 to 0.141. All primary indicators met the inclusion criteria and were retained without modification. The mean importance scores of the second-level indicators ranged from 4.25 (SD 0.45) to 5.00 (SD 0.00), with CVs ranging between 0.000 and 0.220. Detailed results for all indicators are provided in Tables S4 and S5 in Multimedia Appendix 1. No additional feedback or suggestions were provided by the experts. After 2 rounds of the Delphi survey, the evaluation index system for HRSs was established, comprising 5 first-level indicators and 18 second-level indicators (Table 4). Explanations of the indicators are provided in Table 1 and Table S6 in Multimedia Appendix 1.

**Table 4.** Final evaluation index system for health recommender systems developed through a 2-round Delphi consensus study.

Primary indicator	Secondary indicators
Performance	1.1 Accuracy 1.2 Coverage 1.3 Result diversity 1.4 User trust 1.5 Robustness 1.6 Response efficiency
Effectiveness	2.1 Health behavior 2.2 Health outcome 2.3 Quality and efficiency of health services
Safety	3.1 Clinical safety 3.2 Technical safety
Economy	4.1 Patient-level economy 4.2 Institutional-level economy 4.3 Social benefit
Social appropriateness	5.1 Ethicality 5.2 Policy appropriateness 5.3 Acceptability 5.4 Feasibility

### Results of the AHP

The weights assigned to the primary indicators were as follows: 0.136 for performance, 0.193 for effectiveness, 0.289 for safety, 0.132 for economy, and 0.251 for social suitability. The consistency value of 0.032 demonstrates the reliability of the results. Among the secondary indicators, the top 4 weights were attributed to technical safety (0.154), clinical safety (0.135), acceptability (0.065), and policy appropriateness (0.059). All secondary indicators exhibited consistency values below 0.1, further validating the rationality of the weight assignments. The combined weights of the evaluation indicators for HRSs are shown in Table 5.

**Table 5.** Combined weights of primary and secondary evaluation indicators for health recommender systems obtained through the analytic hierarchy process.

Primary indicator (weight) and secondary indicator	Weight	Combined weight
1. Performance	0.136	—^a^
1.1 Accuracy	0.144	0.020
1.2 Coverage	0.151	0.021
1.3 Result diversity	0.157	0.021
1.4 User trust	0.173	0.024
1.5 Robustness	0.183	0.025
1.6 Response efficiency	0.192	0.026
2. Effectiveness	0.193	—
2.1 Health behavior	0.296	0.057
2.2 Health outcome	0.336	0.065
2.3 Quality and efficiency of health services	0.368	0.071
3. Safety	0.289	—
3.1 Clinical safety	0.466	0.135
3.2 Technical safety	0.534	0.154
4. Economy	0.132	—
4.1 Patient-level economy	0.300	0.040
4.2 Institutional-level economy	0.328	0.043
4.3 Social benefit	0.372	0.049
5. Social appropriateness	0.251	—
5.1 Ethicality	0.217	0.054
5.2 Policy appropriateness	0.236	0.059
5.3 Acceptability	0.260	0.065
5.4 Feasibility	0.287	0.072

a Not applicable.

## Discussion

In this study, by integrating literature and policy research with the Delphi expert consultation and AHP methods, an HRS evaluation index system was constructed based on the HTA framework. This system encompasses 5 dimensions—performance, effectiveness, safety, economy, and social appropriateness—comprising a total of 18 secondary assessment indicators.

### Interpretation of Results and Comparison With Previous Research

Existing evaluations of HRSs in most studies predominantly focus on accuracy metrics within the performance dimension, with some studies exhibiting nonstandardized indicator selection and a general lack of user participation in the evaluation process [19]. Even in the few HRS studies that do incorporate user involvement, such participation is typically restricted to assessments of clinical effectiveness, feasibility, or user experience [4]. While these dimensions are important, they alone do not capture the broader clinical, social, and economic implications of deploying RSs in health care settings. General frameworks exist for health IT; however, current evaluation tools tend to be narrowly focused on specific dimensions, such as the user-centric framework proposed by Knijnenburg et al [30]. Some evaluation tools have been developed for DHTs [31-33], such as the Digital Health Technology Assessment developed through the cooperation between the Finnish Coordinating Center for Health Technology Assessment and the University of Oulu’s Faculty of Medicine [33]. This framework was designed to conduct evidence-based reviews of DHTs focusing on dimensions such as product information, technical stability, cost, effectiveness, clinical safety, data security, usability, accessibility, interoperability, and patient and organizational considerations. However, it does not fully address legal, social, and ethical aspects.

Compared with previous research, this work expands the evaluative perspective of HRSs from performance-focused assessment to a holistic, multicriteria framework informed by health technology policy and real-world implementation factors. In terms of core dimensions, our framework aligns with most of the aforementioned evaluation frameworks by addressing safety; effectiveness; economic aspects; organizational implications; and sociocultural, ethical, and legal considerations [31,33]. To address the lack of technical granularity and insufficient practical guidance in existing evaluations of HRSs, we propose an evaluation index system with 2 levels (comprising primary and secondary indicators), providing more specialized and refined assessment support. For example, at the performance level, it specifically incorporates key technical indicators such as recommendation accuracy, content coverage, result diversity, user trust, system robustness, and system response efficiency, tailored to meet the specialized assessment needs of HRSs with regard to algorithmic quality and service efficiency [34]. More importantly, this secondary evaluation index system serves not only as a tool for periodic assessment but also as a driver for the continuous iteration and optimization of HRSs. By integrating quantitative performance data, user feedback, and multidimensional evidence from real-world applications, the evaluation process establishes a closed-loop feedback mechanism.

Quantitative assessment of system performance can identify shortcomings in recommendation accuracy, response speed, coverage, and diversity, thereby providing clear directions and evidence for subsequent technological iterations and optimizations [35]. HRSs are capable of conducting precise analyses based on users’ physiological, psychological, and behavioral data, offering tailored health advice and action plans to facilitate refined health management [30]. However, erroneous recommendations may lead to adverse outcomes, posing risks to patient safety. Therefore, ensuring the effectiveness and safety of recommendation results is crucial for broader promotion and application. Specifically, it is recommended to assess health behaviors and behavioral outcomes through user deployment in real-world application. For example, Bidargaddi et al [17] reported findings from a randomized controlled trial evaluating the efficacy of a guided recommendation service for readily available mobile mental health apps targeting young people. Furthermore, the economic evaluation of HRSs is of paramount importance [36]. Given that the core aim of this technology is to enhance health care efficiency, users can participate in real-world deployment, enabling assessment of whether the system reduces unnecessary medical expenditures, improves diagnostic and treatment quality, and promotes preventive care [37,38]. Such assessments can quantify the cost-effectiveness ratio of the system, providing policymakers with decision-making evidence to determine the feasibility of large-scale promotion and investment. In terms of social appropriateness, HRSs must exhibit good user acceptance, feasibility, and ease of dissemination. Therefore, it is recommended to incorporate user-centered design methods early in the development process. For example, Leng et al [39] used a convergent mixed methods approach to evaluate usability, combining quantitative and qualitative data from diverse users to identify barriers and collaboratively refine system design. Given the unique nature of HRSs as an artificial intelligence technology, the evaluation process must also address ethical issues and regulatory compliance to ensure effective promotion and widespread application [40].

The evaluation index system for HRSs holds significant practical implications for research and development institutions, individual and institutional users, and policymakers in health care management [6]. For organizations involved in the development of HRSs, the system helps development teams in prioritizing core metrics, including accuracy, diversity, real-time performance, and user satisfaction, throughout the technological iteration process [15]. By leveraging the evaluation framework to quantify product performance, the evaluation index system enables the swift identification and resolution of existing issues, thereby significantly enhancing the overall efficacy of HRSs. This provides practical and effective guidance for the design, optimization, and development of HRSs. For health care institutions, HRSs can be leveraged to better serve patients and improve service quality and patient satisfaction while simultaneously reducing medical costs and promoting the efficient allocation of health care resources [41]. This evaluation index system offers clear direction for how institutions can rationally apply and manage such systems in the future. For government agencies, authoritative evaluation results can serve as critical decision-making references when promoting HRSs [42,43]. This aids in formulating policies, regulations, and technical standards that are better aligned with practical needs and specifically targeted to real-world challenges, ensuring the healthy and orderly development of this innovative technology.

### Implications for Clinical Practice and Future Research

First, given the dynamic and evolving trends in user needs and health issues, the evaluation metrics for HRSs will need to be periodically updated to accurately reflect these changes and address diverse health requirements. Second, it is essential to design differentiated evaluation content based on secondary indicators according to the characteristics of different disease profiles or user groups (eg, individuals with chronic conditions) to ensure that the system can deliver personalized recommendations that support individuals in achieving their specific health goals [44]. Finally, further exploration of the application of emerging technologies such as artificial intelligence and blockchain in the evaluation framework is necessary to investigate how to enhance assessment efficiency and accuracy, improve data transparency and security, and provide robust technical support for the continuous optimization of HRSs [45,46].

### Strengths and Limitations

The high expert consensus achieved across Delphi rounds highlights the relevance and acceptability of the proposed indicators. The use of the AHP made the index system clearer and more structured, facilitating its application by decision-makers. As HRSs continue to be integrated into clinical decision support, patient engagement platforms, and digital therapeutics, the evaluation system developed in this study provides a structured foundation for guiding system design, regulatory decision-making, and cross-study comparability.

As a relatively new research field, empirical evidence on the practical implementation of HRSs, particularly systematic deployment, remains limited [47]. In the future, based on updates from empirical research, existing evidence will be integrated to develop specific quantitative methods and evaluation criteria for the HRS evaluation index system. Additionally, the indicators will be further optimized during practical application, continuously improving the evaluation index system for HRSs. It should be noted that the current framework relies heavily on expert input from a specific regional context, primarily China, which may affect its generalizability. Differences in health care systems, cultural norms, and organizational practices across regions may influence the applicability of certain indicators in other settings.

### Conclusions

This study addresses the lack of clear evaluation standards for HRSs by using rigorous scientific methodologies and a systematic development process to construct a comprehensive and structured evaluation index system. In contrast to existing studies that have largely focused on algorithmic performance, this work explicitly incorporates critical but often overlooked dimensions such as safety, economy, and social suitability. By establishing a structured and consensus-based set of criteria, this study addresses a key gap in current HRS evaluation practices and offers a practical foundation for developers, researchers, and policymakers. Future research should apply this framework when designing or deploying HRSs while continuously refining its components to meet emerging technological and ethical challenges in digital health.

## Supplementary material

10.2196/79997 Multimedia Appendix 1 Summary of the studies included in the Delphi process and the importance scores with coefficients of variation for primary indicators across both Delphi rounds.

## Abbreviations

AHP: analytic hierarchy process
Ca: expert judgment coefficient
Cr: expert authority coefficient
Cs: expert familiarity coefficient
CV: coefficient of variation
DHT: digital health technology
HRS: health recommender system
HTA: health technology assessment
RS: recommender system
