# Supplementary material for: Development of an Evaluation Index System for Health Recommender Systems Based on the Health Technology Assessment Framework: Cross-Sectional Delphi Study
Source: JMIR Form Res. 2025 Dec 22;9:e79997. doi: 10.2196/79997 (PMC12721223; doi:10.2196/79997)
Supplement: Multimedia Appendix 1 [file formative-v9-e79997-s001.docx]

Table S1. The basic information of included studies.

| **Author** | **Year** | **Country** | **Primary Indicator** | **Secondary Indicator** |
| --- | --- | --- | --- | --- |
| Agapito G | 2018 | Italy | Performance | Accuracy |
| Asthana S | 2017 | USA | Performance | Accuracy |
| Bravo-Torres JF | 2017 | Spain | Social Appropriateness | Acceptability |
| Buhl M | 2016 | UK | Performance, Social Appropriateness | System Response Efficiency, Acceptability |
| Casino F | 2016 | Greece | Performance | Accuracy, Robustness |
| Chen RC | 2015 | China | Performance | Accuracy |
| Cheng CY | 2017 | China | — | — |
| Elsweiler D | 2015 | UK | Performance | Accuracy, Diversity |
| Halder K | 2017 | Singapore | Performance | Accuracy |
| Ho and Chen | 2009 | Singapore | Effectiveness, Social Appropriateness | Health Behavior, Feasibility |
| Huang YF | 2012 | China | Performance | Content Coverage |
| Jiang H | 2014 | China | Performance, Social Appropriateness | Accuracy, Acceptability |
| Krishna S | 2014 | India | Performance | Accuracy |
| Lafta R | 2015 | China | Performance, Effectiveness | Accuracy, Health Service Efficiency |
| Li J | 2016 | USA | Performance | Accuracy |
| Lima-Medina E | 2014 | Brazil | Performance, Social Appropriateness | Accuracy, Acceptability |
| Lo YW | 2021 | China | Performance | Accuracy |
| Luo Y | 2014 | Canada | Performance, Effectiveness | Prediction Accuracy, Health Behavior Outcomes |
| Mustaqeem A | 2017 | Pakistan | Performance | Accuracy |
| Ntalaperas D | 2015 | Greece | — | — |
| Ooi C | 2015 | Japan | Performance | Accuracy |
| Lin Y | 2012 | Netherlands | Social Appropriateness | Acceptability |
| Pawar KR | 2016 | India | Performance | Accuracy |
| Phanich M | 2010 | Thailand | Social Appropriateness | Satisfaction |
| Radha M | 2016 | Netherlands | Performance | Accuracy |
| Torrent | 2019 | Spain | — | — |
| Wang S | 2016 | China | Performance, Social Appropriateness | Accuracy, User Trust, Acceptability |
| Wongpun S | 2017 | Thailand | Performance | Accuracy |
| Yang L | 2017 | USA | Performance | Accuracy |
| Zaini N | 2012 | Malaysia | Social Appropriateness | Feasibility |
| Zaman N | 2014 | USA | Performance | Accuracy |
| Lin YF | 2014 | China | Performance, Social Appropriateness | Accuracy, Acceptability |
| Wen Z | 2016 | China | Performance | Accuracy |
| Bidargaddi N | 2017 | Australia | Effectiveness | Health Behavior Outcomes |
| Esteban B | 2014 | Spain | Safety, Effectiveness | System Safety, Health Service Efficiency |
| Guo L | 2016 | USA | Performance | Accuracy |
| Wang K | 2019 | China | Performance | Accuracy |
| Huang Y | 2019 | China | Performance | Accuracy |
| Mustaqeem A | 2020 | Pakistan | Performance | Accuracy |
| Kumar A | 2020 | USA | Performance, Effectiveness, Social Appropriateness | Accuracy, Health Service Efficiency, Acceptability |
| Zhao Z | 2020 | Canada | Performance, Social Appropriateness | Accuracy, Acceptability, Data Privacy Protection, User Satisfaction |
| Brenas JH | 2019 | USA | Social Appropriateness | Acceptability |
| Wang Z | 2020 | China | Performance | Accuracy |
| Gannod GC | 2019 | USA | Performance | Accuracy |
| Mantey EA | 2021 | UK | Performance | Accuracy |
| Chen J | 2021 | USA | Effectiveness | Health Behavior Outcomes |
| Li X | 2021 | China | Performance | Accuracy |
| Pecune F | 2021 | USA | Performance, Social Appropriateness | Accuracy, Acceptability |
| Granda M | 2022 | Spain | Performance | Accuracy |
| Jung YL | 2022 | South Korea | Performance | Accuracy |
| Shao M | 2022 | China | Effectiveness | Health Behavior Outcomes |
| Torrent-Fontbona F | 2019 | Spain | Effectiveness | Quality of Life |
| Gutiérrez F | 2018 | Spain | Performance | Accuracy, Diversity |
| Li J | 2016 | USA | Performance | Accuracy |
| Wang T | 2021 | Finland | Economic Efficiency | Cost-Effectiveness |
| Liu C | 2022 | UK | Safety | Adverse Event Rate, System Safety |
| Hors-Fraile S | 2018 | Spain | Social Appropriateness, Effectiveness | Data Privacy Security, Acceptability, Health Behavior Outcomes, Quality of Life |
| Avari P | 2021 | UK | Safety, Effectiveness | Adverse Event Rate, System Safety, Health Behavior Outcomes, Quality of Life |
| Esteban B | 2014 | Spain | Safety, Performance | System Safety, User Trust |
| Barros M | 2021 | UK | Performance | Accuracy |
| Chiang J | 2020 | USA | Effectiveness, Social Appropriateness | Health Service Efficiency, Acceptability |
| Ueta T | 2011 | USA | Performance | Precision |
| Yang L | 2016 | USA | Performance, Social Appropriateness | Precision, Acceptability |
| Zhao Z | 2020 | Canada | Effectiveness | Health Behavior Outcomes |
| Lafta R | 2015 | Australia | Economic Efficiency | Institutional Cost-Effectiveness |
| Medina EL | 2016 | Brazil | Social Appropriateness | Acceptability |
| Maguire R | 2015 | UK | Social Appropriateness | Acceptability, Feasibility |
| Jacobs M | 2019 | USA | Social Appropriateness | Acceptability |
| Kanera IM | 2017 | Netherlands | Effectiveness, Safety | Health Behavior Outcomes, Adverse Event Rate |
| Mooney KH | 2016 | USA | Effectiveness | Health Behavior Outcomes |
| Sanchez Bocanegra CL | 2017 | Spain | Performance | Accuracy, Robustness |
| Huang Yujing | 2016 | China | Performance | Accuracy |
| Wang Kai | 2019 | China | Performance | Accuracy |
| Li Yueyan | 2019 | China | Performance | Accuracy |
| Wang Yue | 2019 | China | Performance | Accuracy |
| Gao Shan | 2019 | China | Performance | Accuracy |
| Wang Qun | 2016 | China | Performance | Accuracy |

[1] Agapito G, Simeoni M, Calabrese B, et al. DIETOS: A dietary recommender system for chronic diseases monitoring and management [J]. Computer methods and programs in biomedicine, 2018, 153: 93-104.

[2] Asthana S, Megahed A, Strong R. A recommendation system for proactive health monitoring using IoT and wearable technologies[C]//2017 IEEE international conference on AI & mobile services (AIMS). IEEE, 2017: 14-21.

[3] Bravo-Torres JF, Jorge O. Ordoñez-Ordoñez, Gallegos-Segovia PL, et al. A context-aware platform for comprehensive care of elderly people: Proposed architecture [C]. Electrical, Electronics Engineering, Information & Communication Technologies. IEEE, 2017.

[4] Buhl M, Famulare J, Glazier C, et al. Optimizing multi-channel health information delivery for behavioral change[C]//2016 IEEE Systems and Information Engineering Design Symposium (SIEDS). IEEE, 2016: 130-135.

[5] Casino F, Patsakis C, Batista E, et al. Healthy Routes in the Smart City: A Context-Aware Mobile Recommender [J]. IEEE Software, 2017, 34(6):42-7.

[6] Chen R C, Ting Y H, Chen J K, et al. The nutrients of chronic diet recommended based on domain ontology and decision tree[C]//2015 conference on technologies and applications of artificial intelligence (TAAI). IEEE, 2015: 289-295.

[7] Cheng CY, Hong YY. Kiasu and Creativity in Singapore: An Empirical Test of the Situated Dynamics Framework [J]. Management and Organization Review, 2017, 13(4): 871-94.

[8] Elsweiler D, Harvey M. Towards automatic meal plan recommendations for balanced nutrition[C]//Proceedings of the 9th ACM Conference on Recommender Systems. 2015: 313-316.

[9] Halder K, Kan M Y, Sugiyama K. Health forum thread recommendation using an interest aware topic model[C]//Proceedings of the 2017 ACM on Conference on Information and Knowledge Management. 2017: 1589-1598.

[10] Ho T C T, Chen X. ExerTrek: A portable handheld exercise monitoring, tracking and recommendation system[C]//2009 11th International Conference on e-Health Networking, Applications and Services (Healthcom). IEEE, 2009: 84-88.

[11] Huang Y F, Liu P, Pan Q, et al. A doctor recommendation algorithm based on doctor performances and patient preferences[C]//2012 International Conference on Wavelet Active Media Technology and Information Processing (ICWAMTIP). IEEE, 2012: 92-95.

[12] Jiang H, Xu W. How to find your appropriate doctor: An integrated recommendation framework in big data context[C]//2014 IEEE Symposium on Computational Intelligence in Healthcare and e-health (CICARE). IEEE, 2014: 154-158.

[13] Krishna S, Swagath S, Valliyammai C. A trust enhanced Recommender System for medicare applications[C]//2014 Sixth International Conference on Advanced Computing (ICoAC). IEEE, 2014: 324-328.

[14] Lafta R, Zhang J, Tao X, et al. An intelligent recommender system based on short-term risk prediction for heart disease patients[C]//2015 IEEE/WIC/ACM International Conference on Web Intelligence and Intelligent Agent Technology (WI-IAT). IEEE, 2015, 3: 102-105.

[15] Li J, Kong J. Cell phone-based diabetes self-management and social networking system for American Indians[C]//2016 IEEE 18th International Conference on e-Health Networking, Applications and Services (Healthcom). IEEE, 2016: 1-6.

[16] Lima-Medina E, Loques O, Mesquita C. " Minha Saude" a healthcare social network for patients with cardiovascular diseases[C]//2014 IEEE 3nd International Conference on Serious Games and Applications for Health (SeGAH). IEEE, 2014: 1-7.

[17] Lo Y W, Zhao Q, Ting YH, et al. Automatic generation and recommendation of recipes based on outlier analysis[C]//. IEEE International Conference on Awareness Science & Technology. IEEE, 2015.

[18] Luo Y, Ling C, Schuurman J, et al. GlucoGuide: an intelligent type-2 diabetes solution using data mining and mobile computing[C]//2014 IEEE International Conference on Data Mining Workshop. IEEE, 2014: 748-752.

[19] Mustaqeem A, Anwar SM, Khan AR, et al. A statistical analysis based recommender model for heart disease patients [J]. International journal of medical informatics, 2017, 108: 134-45.

[20] Ntalaperas D, Bothos E, Perakis K, et al. DISYS: An intelligent system for personalized nutritional recommendations in restaurants[C]//Proceedings of the 19th Panhellenic Conference on Informatics. 2015: 382-387.

[21] Ooi A, Iiba T, Takano K. Ingredient substitute recommendation for allergy-safe cooking based on food context[C]//2015 IEEE Pacific Rim Conference on Communications, Computers and Signal Processing (PACRIM). IEEE, 2015: 444-449.

[22] Lin Y, Jessurun J, De Vries B, et al. Motivate: Towards context-aware recommendation mobile system for healthy living[C]//2011 5th International Conference on Pervasive Computing Technologies for Healthcare (PervasiveHealth) and Workshops. IEEE, 2011: 250-253.

[23] Pawar K R, Ghorpade T, Shedge R. Constraint based recipe recommendation using forward checking algorithm[C]//2016 International Conference on Advances in Computing, Communications and Informatics (ICACCI). IEEE, 2016: 1474-1478.

[24] Phanich M, Pholkul P, Phimoltares S. Food recommendation system using clustering analysis for diabetic patients[C]//2010 international conference on information science and applications. IEEE, 2010: 1-8.

[25] Radha M, Willemsen M C, Boerhof M, et al. Lifestyle recommendations for hypertension through rasch-based feasibility modeling[C]//Proceedings of the 2016 Conference on User Modeling Adaptation and Personalization. 2016: 239-247.

[26] Torrent-Fontbona F, Lopez B. Personalized Adaptive CBR Bolus Recommender System for Type 1 Diabetes [J]. IEEE Journal of Biomedical and Health Informatics, 2019, 23(1): 387-94.

[27] Wang SL, Chen YL, Kuo A MH, et al. Design and evaluation of a cloud-based Mobile Health Information Recommendation system on wireless sensor networks [J]. Computers & Electrical Engineering, 2016, 49: 221-35.

[28] Wongpun S, Guha S. Elderly care recommendation system for informal caregivers using case-based reasoning[C]//2017 IEEE 2nd Advanced Information Technology, Electronic and Automation Control Conference (IAEAC). IEEE, 2017: 548-552.

[29] Yang L, Hsieh CK, Yang H, et al. Yum-Me: A Personalized Nutrient-Based Meal Recommender System [J]. ACM transactions on information systems, 2017, 36(1):1-31.

[30] Zaini N, Latip MFA, Omar H, et al. Online personalized audio therapy recommender based on community ratings[C]//. IEEE Symposium on Computer Applications & Industrial Electronics. IEEE, 2012.

[31] Li Q, Li J, Wang H, et al. Semantics-enhanced privacy recommendation for social networking sites[C]//2011IEEE 10th International Conference on Trust, Security and Privacy in Computing and Communications. IEEE, 2011: 226-233.

[32] Lin YF, Shie HH, Yang YC, et al. Design of a real-time and continua-based framework for care guideline recommendations [J]. International journal of environmental research and public health, 2014, 11(4): 4262-79.

[33] El-Gayar O, Timsina P, Nawar N, et al. Mobile applications for diabetes self-management: status and potential [J]. Journal of diabetes science and technology, 2013, 7(1): 247-62.

[34] Bidargaddi N, Musiat P, Winsall M, et al. Efficacy of a Web-Based Guided Recommendation Service for a Curated List of Readily Available Mental Health and Well-Being Mobile Apps for Young People: Randomized Controlled Trial [J]. Journal of medical Internet research, 2017, 19(5): e141.

[35] Esteban, B, Tejeda-Lorente A, Porcel C, et al. TPLUFIB-WEB: A fuzzy linguistic Web system to help in the treatment of low back pain problems [J]. Knowledge-Based Systems, 2014, 67:429-38.

[36] Guo L, Jin B, Yao C, et al. Which Doctor to Trust: A Recommender System for Identifying the Right Doctors [J]. Journal of medical Internet research, 2016, 18(7): e186.

[37] Huang Yujing. Design and Implementation of a Personalized Health Information Service System [D]. Beijing: Beijing University of Posts and Telecommunications, 2019.

[38] Wang Kai. Research and Implementation of a Personalized Health Knowledge Recommendation System [D]. Wuhan: Hubei University of Technology, 2019.

[39] Mustaqeem A, Anwar SM, Majid M. A modular cluster based collaborative recommender system for cardiac patients [J]. Artificial intelligence in medicine, 2020, 102: 101761.

[40] Kumar A, Aikens RC, Hom J, et al. OrderRex clinical user testing: a randomized trial of recommender system decision support on simulated cases [J]. Journal of the American Medical Informatics Association, 2020, 27(12): 1850-9.

[41] Zhao Z, Arya A, Orji R, et al. Effects of a Personalized Fitness Recommender System Using Gamification and Continuous Player Modeling: System Design and Long-Term Validation Study [J]. JMIR Serious Games, 2020, 8(4): e19968.

[42] Brenas JH, Shin EK, Shaban-Nejad A. A Hybrid Recommender System to Guide Assessment and Surveillance of Adverse Childhood Experiences [J]. Studies in health technology and informatics, 2019, 262: 332-5.

[43] Wang Z, Huang H, Cui L, et al. Using Natural Language Processing Techniques to Provide Personalized Educational Materials for Chronic Disease Patients in China: Development and Assessment of a Knowledge-Based Health Recommender System [J]. JMIR Medical Informatics, 2020, 8(4): e17642.

[44] Gannod GC, Abbott KM, Van Haitsma K, et al. A Machine Learning Recommender System to Tailor Preference Assessments to Enhance Person-Centered Care Among Nursing Home Residents [J]. Gerontologist, 2019, 59(1): 167-76.

[45] Mantey EA, Zhou C, Anajemba JH, et al. Blockchain-Secured Recommender System for Special Need Patients Using Deep Learning [J]. Frontiers in public health, 2021, 9: 737269.

[46] Chen J, Houston TK, Faro JM, et al. Evaluating the use of a recommender system for selecting optimal messages for smoking cessation: patterns and effects of user-system engagement [J]. BMC Public Health, 2021, 21(1): 1749.

[47] Li X, Ma D, Ren Y, et al. Large-scale Prediction of Drug-Protein Interactions Based on Network Information [J]. Current computer-aided drug design, 2022, 18(1): 64-72.

[48] Pecune F, Callebert L, Marsella S. Designing Persuasive Food Conversational Recommender Systems With Nudging and Socially-Aware Conversational Strategies [J]. Frontiers in robotics and AI, 2021, 8: 733835.

[49] Granda Morales LF, Valdiviezo-Diaz P, Reátegui R, et al. Drug Recommendation System for Diabetes Using a Collaborative Filtering and Clustering Approach: Development and Performance Evaluation [J]. Journal of medical Internet research, 2022, 24(7): e37233.

[50] Jung YL, Yoo HS, Hwang J. Artificial intelligence-based decision support model for new drug development planning [J]. Expert systems with applications, 2022, 198: 116825.

[51] Shao M, Jiang L, Meng Z, et al. Computational Drug Repurposing Based on a Recommendation System and Drug-Drug Functional Pathway Similarity [J]. Molecules, 2022, 27(4): 1404.

[52] Hors-Fraile S, Schneider F, Fernandez-Luque L, et al. Tailoring motivational health messages for smoking cessation using an mHealth recommender system integrated with an electronic health record: a study protocol [J]. BMC Public Health, 2018, 18(1): 698.

[53] Torrent-Fontbona F, Lopez B. Personalized Adaptive CBR Bolus Recommender System for Type 1 Diabetes [J]. IEEE Journal of Biomedical and Health Informatics, 2019, 23(1): 387-94.

[54] Zemplényi A, Tachkov K, Balkanyi L, et al. Recommendations to overcome barriers to the use of artificial intelligence-driven evidence in health technology assessment [J]. Frontiers in public health, 2023, 11: 1088121.

[55] Li J, Kong J. Cell phone-based diabetes self-management and social networking system for American Indians[C]//2016 IEEE 18th International Conference on e-Health Networking, Applications and Services (Healthcom). IEEE, 2016: 1-6.

[56] Wang T, Szedmak S, Wang H, et al. Modeling drug combination effects via latent tensor reconstruction [J]. Bioinformatics, 2021, 37(Suppl_1): i93-i101.

[57] Liu C, Avari P, Leal Y, et al. A Modular Safety System for an Insulin Dose Recommender: A Feasibility Study [J]. Journal of diabetes science and technology, 2020, 14(1): 87-96.

[58] Hors-Fraile S, Schneider F, Fernandez-Luque L, et al. Tailoring motivational health messages for smoking cessation using an mHealth recommender system integrated with an electronic health record: a study protocol [J]. BMC Public Health, 2018, 18(1): 698.

[59] Esteban B, Tejeda-Lorente, Porcel C, et al. TPLUFIB-WEB: A fuzzy linguistic Web system to help in the treatment of low back pain problems [J]. Knowledge-Based Systems, 2014, 67:429-38

[60] Barros M, Ruas P, Sousa D, et al. COVID-19 recommender system based on an annotated multilingual corpus [J]. Genomics & informatics, 2021, 19(3): e24.

[61] Chiang J, Kumar A, Morales D, et al. Physician Usage and Acceptance of a Machine Learning Recommender System for Simulated Clinical Order Entry [J]. AMIA Joint Summits on Translational Science proceedings AMIA Joint Summits on Translational Science, 2020: 89-97.

[62] Ueta T, Iwakami M, Ito T. Implementation of a goal-oriented recipe recommendation system providing nutrition information[C]//2011 International Conference on Technologies and Applications of Artificial Intelligence. IEEE, 2011: 183-188.

[63] Yang L, Hsieh CK, Yang H, et al. Yum-Me: A Personalized Nutrient-Based Meal Recommender System [J]. ACM transactions on information systems, 2017, 36(1): 7.

[64] Zhao Z, Arya A, Orji R, et al. Effects of a Personalized Fitness Recommender System Using Gamification and Continuous Player Modeling: System Design and Long-Term Validation Study [J]. JMIR Serious Games, 2020, 8(4): e19968.

[65] Lafta R, Zhang J, Tao X, et al. An intelligent recommender system based on short-term risk prediction for heart disease patients[C]//2015 IEEE/WIC/ACM International Conference on Web Intelligence and Intelligent Agent Technology (WI-IAT). IEEE, 2015, 3: 102-105.

[66] Medina EL, Mesquita CT, Filho OL. Healthcare Social Networks for Patients with Cardiovascular Diseases and Recommendation Systems[J]. International Journal of Cardiovascular Sciences, 2015, 28(6).

[67] Maguire R, Ream E, Richardson A, et al. Development of a novel remote patient monitoring system: the advanced symptom management system for radiotherapy to improve the symptom experience of patients with lung cancer receiving radiotherapy [J]. Cancer nursing, 2015, 38(2): E37-47.

[68] Jacobs M, Hopkins J, Mumber M, et al. Usability Evaluation of an Adaptive Information Recommendation System for Breast Cancer Patients [J]. AMIA Annual Symposium proceedings AMIA Symposium, 2019: 494-503.

[69] Kanera IM, Willems RA, Bolman CA, et al. Long-term effects of a web-based cancer aftercare intervention on moderate physical activity and vegetable consumption among early cancer survivors: a randomized controlled trial [J]. The international journal of behavioral nutrition and physical activity, 2017, 14(1): 19.

[70] Mooney KH, Beck SL, Wong B, et al. Automated home monitoring and management of patient-reported symptoms during chemotherapy: results of the symptom care at home RCT [J]. Cancer medicine, 2017, 6(3): 537-46.

[71] Sanchez Bocanegra CL, Sevillano Ramos JL, Rizo C, et al. HealthRecSys: A semantic content-based recommender system to complement health videos [J]. BMC medical informatics and decision making, 2017, 17(1): 63.

[72] Huang Yujing. Design and Implementation of a Personalized Health Information Service System [D]. Beijing: Beijing University of Posts and Telecommunications, 2019.

[73] Wang Kai. Research and Implementation of a Personalized Health Knowledge Recommendation System [D]. Wuhan: Hubei University of Technology, 2019.

[74] Li Yueyan. Research on Doctor Recommendation Based on Online Consultation Platforms [D]. Wuhan: Central China Normal University, 2019.

[75] Wang Yue. Design and Implementation of a Health Diet Recommendation System Based on Multi-Features [D]. Chongqing: Chongqing University of Posts and Telecommunications, 2021.

[76] Gao Shan. Research on Collaborative Filtering Recommendation Algorithm Based on Multiple User Behavior Feedback and Its Application in Health Service Platforms [D]. Zhengzhou: Zhengzhou University, 2019.

[77] Wang Qun. Research and Application of a Health Knowledge Recommendation System Based on Collaborative Filtering [D]. Chengdu: University of Electronic Science and Technology of China, 2016.

Table S2. Importance scores and coefficients of variation for primary indicators in Round 1 of the Delphi survey.

| Primary Indicator | Mean | Standard Deviation | Coefficient of Variation | Decision |
| --- | --- | --- | --- | --- |
| Performance | 4.44 | 0.69 | 0.155 | Retained |
| Effectiveness | 4.83 | 0.83 | 0.172 | Retained |
| Safety | 5.00 | 0.00 | 0.000 | Retained |
| Economy | 4.39 | 0.59 | 0.134 | Retained |
| Social Appropriateness | 4.50 | 0.60 | 0.133 | Retained |

Table S3. Importance scores and coefficients of variation for secondary indicators in Round 1 of the Delphi survey.

| Primary Indicator | Secondary Indicator | Mean | Standard Deviation | Coefficient of Variation | Decision |
| --- | --- | --- | --- | --- | --- |
| 1.Performance | 1.1 Accuracy | 4.83 | 0.37 | 0.077 | Retained |
|  | 1.2 Coverage | 4.22 | 0.53 | 0.126 | Retained |
|  | 1.3 Result Diversity | 4.17 | 0.60 | 0.144 | Retained |
|  | 1.4 User Trust | 4.83 | 0.37 | 0.077 | Retained |
|  | 1.5 Robustness | 4.33 | 0.75 | 0.173 | Retained |
|  |  |  |  |  |  |
| 2. Effectiveness | 2.1 Health Behavior Outcomes | 4.94 | 0.23 | 0.046 | Retained |
|  | 2.2 Quality of Life Impact | 4.94 | 0.23 | 0.046 | Deleted |
|  | 2.3 Health Service Efficiency | 4.61 | 0.59 | 0.128 | Revised |
|  |  |  |  |  |  |
| 3. Safety | 3.1 Clinical Safety | 5.00 | 0.00 | 0.000 | Retained |
|  | 3.2 Technical Application Safety | 4.72 | 0.45 | 0.095 | Retained |
|  |  |  |  |  |  |
| 4.Economy | 4.1 Patient Cost-Effectiveness | 4.39 | 0.49 | 0.112 | Revised |
|  | 4.2 Institutional Cost-Effectiveness | 4.50 | 0.50 | 0.111 | Revised |
|  | 4.3 Social Benefits | 4.28 | 0.65 | 0.152 | Retained |
|  |  |  |  |  |  |
| 5. Social Appropriateness | 5.1 Data Privacy Protection | 4.72 | 0.45 | 0.095 | Deleted |
|  | 5.2 Legal and Policy Compliance | 4.35 | 0.68 | 0.156 | Revised |
|  | 5.3 Acceptability | 4.44 | 0.68 | 0.153 | Revised |

Table S4. Importance scores and coefficients of variation for primary indicators in Round 2 of the Delphi survey.

| Primary Indicator | Mean | Standard Deviation | Coefficient of Variation | Adjustment |
| --- | --- | --- | --- | --- |
| Performance | 4.88 | 0.69 | 0.141 | Retained |
| Effectiveness | 4.94 | 0.37 | 0.074 | Retained |
| Safety | 4.88 | 0.00 | 0.000 | Retained |
| Economy | 4.31 | 0.59 | 0.137 | Retained |
| Social Appropriateness | 4.63 | 0.6 | 0.130 | Retained |

Table S5. Importance scores and coefficients of variation for secondary indicators in Round 2 of the Delphi survey.

| Primary Indicator | Secondary Indicator | Mean | Standard Deviation | Coefficient of Variation | Adjustment |
| --- | --- | --- | --- | --- | --- |
| 1. Performance | 1.1 Accuracy | 4.69 | 0.60 | 0.128 | Retained |
|  | 1.2 Coverage | 4.50 | 0.52 | 0.116 | Retained |
|  | 1.3 Result Diversity | 4.63 | 0.50 | 0.108 | Retained |
|  | 1.4 User Trust | 4.88 | 0.34 | 0.070 | Retained |
|  | 1.5 Robustness | 4.81 | 0.40 | 0.083 | Retained |
|  | 1.6 Response Efficiency | 4.31 | 0.95 | 0.220 | Retained |
| 2. Effectiveness | 2.1 Health Behavior | 5.00 | 0.00 | 0.000 | Retained |
|  | 2.2 Health Outcome | 4.94 | 0.25 | 0.051 | Retained |
|  | 2.3 Quality and Efficiency of Health Services | 4.63 | 0.50 | 0.110 | Retained |
| 3. Safety | 3.1 Clinical Safety | 5.00 | 0.00 | 0.000 | Retained |
|  | 3.2 Technical Safety | 4.75 | 0.45 | 0.095 | Retained |
| 4. Economy | 4.1 Patient-level Economy | 4.63 | 0.50 | 0.108 | Retained |
|  | 4.2 Institutional-level Economy | 4.56 | 0.63 | 0.138 | Retained |
|  | 4.3 Social Benefit | 4.25 | 0.45 | 0.106 | Retained |
| 5.Social Appropriateness | 5.1 Ethicality | 4.94 | 0.25 | 0.051 | Retained |
|  | 5.2 Policy Appropriateness | 4.75 | 0.45 | 0.096 | Retained |
|  | 5.3 Acceptability | 4.81 | 0.40 | 0.083 | Retained |
|  | 5.4 Feasibility | 4.75 | 0.45 | 0.095 | Retained |

Table S6. The explanation of the indicators.

| **Primary Indicator** | **Secondary Indicator** | **Indicator Explanation** | **Indicator Direction** |
| --- | --- | --- | --- |
| 1. Performance | 1.1 Accuracy | The degree to which the system’s recommendations correctly match the user’s actual needs, preferences, or health status, as reflected by the precision of the recommended items or decisions. | Gradual improvement |
|  | 1.2 Coverage | The proportion of recommended content relative to the total available items, indicating the breadth of the system’s recommendation coverage. | Gradual improvement |
|  | 1.3 Result Diversity | The degree of variation within the content recommended to individual users or user groups. | Gradual improvement |
|  | 1.4 User Trust | The level of user confidence in the system and their willingness to follow its recommendations. | Gradual improvement |
|  | 1.5 Robustness | Refers to the system’s ability to maintain stable, accurate, and effective recommendations under diverse or dynamically changing conditions. | Gradual improvement |
|  | 1.6 Response Efficiency | The duration from a user's recommendation request to the system's delivery of results. | Gradual reduction |
| 2. Effectiveness | 2.1 Health Behavior | The influence of the system on user behaviors, such as physical activity, dietary habits, or agitation. | Monitoring and comparison |
|  | 2.2 Health Outcome | The impact of the system on health-related outcomes, including physical and biochemical indicators, social support, and psychological well-being. | Monitoring and comparison |
|  | 2.3 Quality and Efficiency of Health Services | The extent to which the system enhances the quality or efficiency of health services for stakeholders (e.g., patients, physicians, nurses, or family members). | Monitoring and comparison |
| 3. Safety | 3.1 Clinical Safety | The incidence of adverse events or serious adverse events during the system's use. | Monitoring and comparison |
|  | 3.2 Technical Safety | The system's safety in terms of network security, algorithm integrity, data privacy, management protocols, and server operations. | Monitoring and comparison |
| 4. Economy | 4.1 Patient-level Economy | The financial burden and cost-effectiveness for patients utilizing the system. | Gradual improvement |
|  | 4.2 Institutional-level Economy | The economic benefits that the health recommender system brings to healthcare institutions, such as reduced operational costs, improved resource utilization, or enhanced service efficiency. | Gradual improvement |
|  | 4.3 Social Benefit | The system’s contribution to broader societal, economic, and healthcare sector development. | Gradual improvement |
| 5.Social Appropriateness | 5.1 Ethicality | The system's adherence to established medical ethical standards. | Monitoring and comparison |
|  | 5.2 Policy Appropriateness | The degree to which the system complies with national laws, policies, and regulatory frameworks. | Monitoring and comparison |
|  | 5.3 Acceptability | The level of user acceptance and satisfaction with the system. | Gradual improvement |
|  | 5.4 Feasibility | The extent to which the system's services meet user needs and are readily available. | Gradual improvement |
